# Efficacy of Everolimus Low-Dose Treatment for Cardiac Rhabdomyomas in Neonatal Tuberous Sclerosis: Case Report and Literature Review

**DOI:** 10.3390/pediatric13010015

**Published:** 2021-03-01

**Authors:** Luisa Federica Nespoli, Elena Albani, Carla Corti, Luigina Spaccini, Enrico Alfei, Irene Daniele, Gian Vincenzo Zuccotti, Gianluca Lista, Valeria Calcaterra, Savina Mannarino

**Affiliations:** 1 Pediatric Cardiology Unit, “Vittore Buzzi” Children’s Hospital, 20154 Milano, Italy; luisa.nespoli@asst-fbf-sacco.it (L.F.N.); carla.corti@asst-fbf-sacco.it (C.C.); 2 Department of Pediatrics, “Vittore Buzzi” Children’s Hospital, 20154 Milano, Italy; elena.albani1@gmail.com (E.A.); gianvincenzo.zuccotti@unimi.it (G.V.Z.); valeria.calcaterra@unipv.it (V.C.); 3 Clinical Genetics Unit, Department of Obstetrics and Gynecology, “V. Buzzi” Children’s Hospital, University of Milano, 20154 Milano, Italy; luigina.spaccini@asst-fbf-sacco.it; 4 Pediatric Neurology Unit, “V. Buzzi” Children’s Hospital, Milano, 20154 Milano, Italy; enrico.alfei@asst-fbf-sacco.it; 5 Neonatal Pathology and Neonatal Intensive Care Unit, “V. Buzzi” Children’s Hospital, University of Milano, 20154 Milano, Italy; irene.daniele@asst-fbf-sacco.it (I.D.); gianluca.lista@asst-fbf-sacco.it (G.L.); 6 Department of Biomedical and Clinical Science “L. Sacco”, University of Milano, 20157 Milano, Italy; 7 Pediatric and Adolescent Unit, Department of Internal Medicine, University of Pavia, 27100 Pavia, Italy

**Keywords:** cardiac rhabdomyoma, tuberous sclerosis, everolimus, neonate, low dose

## Abstract

*Background*: Cardiac rhabdomyomas (CRs) are the most common cardiac tumors in newborns. Approximately 80–90% of cases are associated with tuberous sclerosis complex (TSC). In selective cases, Everolimus has resulted in a remarkable tumoral regression effect in children with TS. The optimal dosage for neonates is still unknown. *Case presentation*: We describe the use of Everolimus in a neonate with multiple biventricular CRs, causing subaortic obstruction, in which a low-dose treatment (0.1 mg/die), in an effort to maintain serum trough levels of 3–7 ng/mL, was successfully used off-label, without adverse effects. *Conclusions*: We showed that a low-dose Everolimus regimen may be an effective and safe treatment for CR regression in TS neonates, when the minimum therapeutic range was maintained.

## 1. Introduction

Tuberous sclerosis complex (TSC) is a rare autosomal dominant genetic disorder, characterized by over-activation of the mammalian target of the rapamycin (mTOR) pathway [1,2]. Recent studies estimate a frequency of 1/6000–1/10,000 of live births and a population prevalence of around 1/20,000 [1,2]. This genetic disorder leads to the growth of hamartomas in multiple organs, such as the skin, central nervous system, angiofibromas, renal angiomyolipoma, and cardiac rhabdomyomas (CRs) [2,3]. 

CRs account for up to 90% of cardiac tumors in the pediatric population under the age of 1 and are associated with TSC in 60–80% of the cases [3,4]. 

Everolimus is a serine–threonine kinase mammalian target of rapamycin inhibitor, which is able to inhibit growth-driven cell proliferation [5,6]. Everolimus is currently approved by the Food and Drug Administration (FDA) and the European Medicines Agency for TSC-associated partial-onset seizures, subependymal giant cell astrocytoma, and renal angiomyolipoma in children [7,8]. Everolimus has been successfully used off-label in pediatric case reports of symptomatic CRs associated with TSC [7,8,9]. The optimal dosage for neonates is still unknown and is currently under debate [3,5,10,11,12,13,14,15,16,17,18,19,20,21].

We described the use of Everolimus in a neonate with multiple biventricular CRs, in which a low-dose treatment was successfully adopted, maintaining a minimum therapeutic range. A literature review of the low-dose drug treatment during the neonatal period was also carried out.

## 2. Case Presentation

A full-term male infant was born at 40 + 4 gestational weeks, with a birth weight of 4660 g and a normal perinatal adaptation. No remarkable family history for neurologic pathologies or symptoms suggestive of TSC was recorded. 

**Figure 1:**
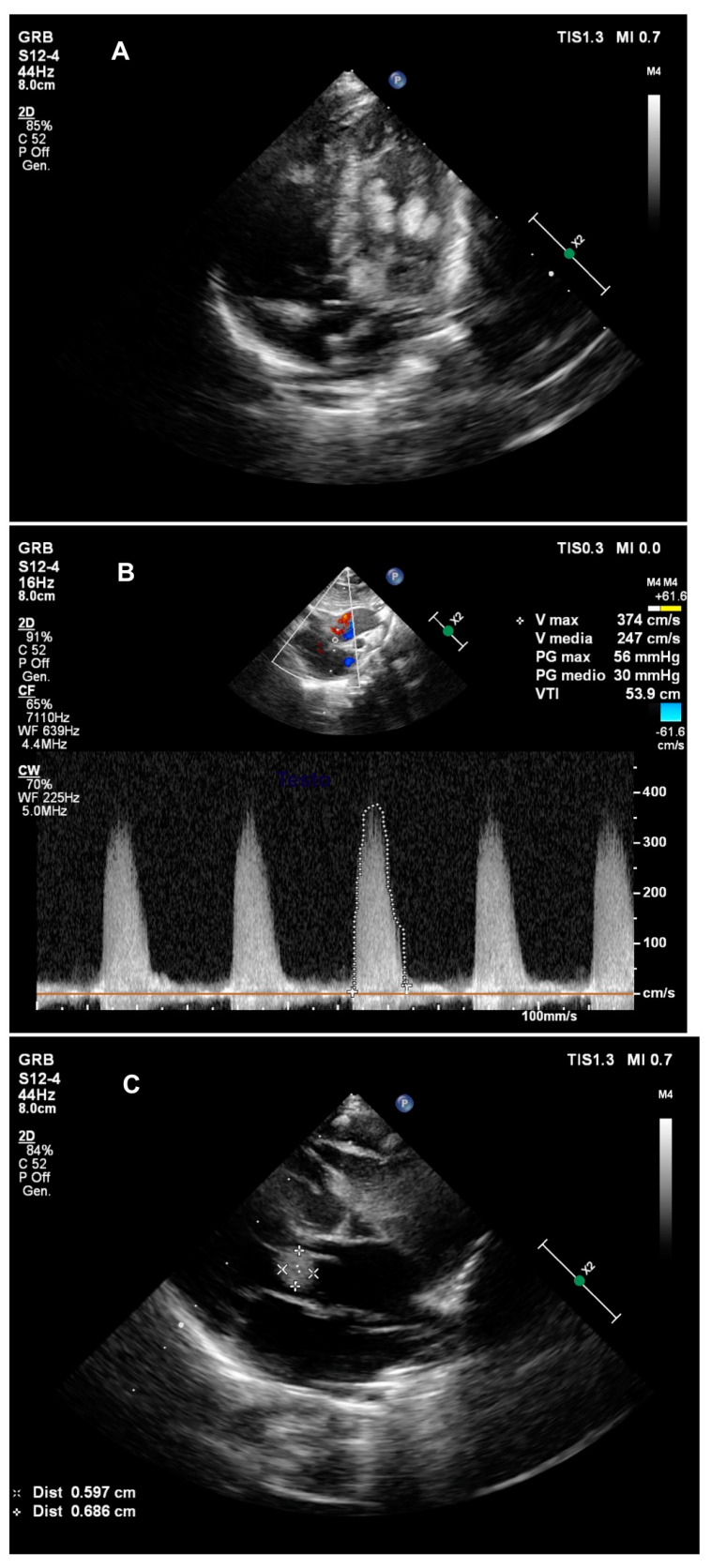
Echocardiogram on the first day of life. Panel (**A**): Left ventricle rhabdomyomas; Panel (**B**): Elevated transaortic gradient; Panel (**C**): Subaortic mass.

He underwent echocardiography on the first day of life for the presence of a cardiac murmur 3/6 left (L) on the precordium. At the first cardiologic visit, the baby revealed good clinical conditions, with normal blood pressure and heart rate of 142 bpm. ECG readings were normal. The echocardiographic evaluation showed normal biventricular dimensions and systolic function, with multiple highly echogenic cardiac masses. In particular, the following lesions were located: One in the medium-apical portion of the interventricular septum (7 × 2 mm); three multi-lobular shaped lesions on the mitral valve chordae (7 × 3 mm; 8.5 × 3.5 mm; 16 × 4 mm), leading to a mild acceleration of left ventricular output (v max 2.4 m/s); one in the left ventricular apex (8.5 × 2.5 mm); one in the mid-apical segment of the right ventricle area (0.6 cm^2^, 7 × 8 mm in apical view); and one in the subaortic region (10 × 10 mm, area 0.7 cm^2^ in apical view), originating from the outlet septum, extending towards the mitral valve, with a tiny 1.5 mm space for blood passage between the mass and the non-coronary aortic cusp, leading to an elevated pressure gradient in the left outflow tract (grad max/medium 60/40 mmHg), Figure 1. 

A diagnosis of multiple CRs was made. The baby was transferred to the Neonatal Intensive Care Unit for continuous cardiac monitoring. In the hypothesis of CRs associated with TSC, a cerebral MRI, abdominal ultrasound, EEG, eye and dermatological examinations were performed, all of which resulted negative. The genetic screening of the proband and parents revealed a de novo pathognomonic mutation in the TSC2 gene. 

The baby remained asymptomatic, with excellent weight gain and no feeding difficulties. ECG monitoring revealed no cardiac arrhythmia. Nonetheless, due to the location and number of cardiac tumors, we decided to begin a low-dosage administration of propanolol (1.5 mg/kg/die orally), which was well tolerated. 

Due to the severe gradient in the left ventricular outlet, the clinical case was discussed with cardiac surgeons. Considering the high risk of complications, they decided not to perform resection of the subaortic mass. 

After a multidisciplinary consultation meeting with cardiologists, neonatologists, a neurologist, and a geneticist, the parents agreed to begin the off-label Everolimus therapy. The baby was given Everolimus, 0.1 mg/die per os, starting at 26 days. 

The serum Everolimus level 5 days after the initial dose was 4.5 ng/mL, the baby was well and the intensity of the cardiac murmur had dropped to 1-2/6 L. The CRs had already begun to shrink: The subaortic mass shrank to 6.5 × 9 mm, with a 2.7 mm space between the mass and the aortic wall; the transaortic pressure gradient halved to 24/14 mmHg, with no acceleration of the left ventricular output.

**Figure 2:**
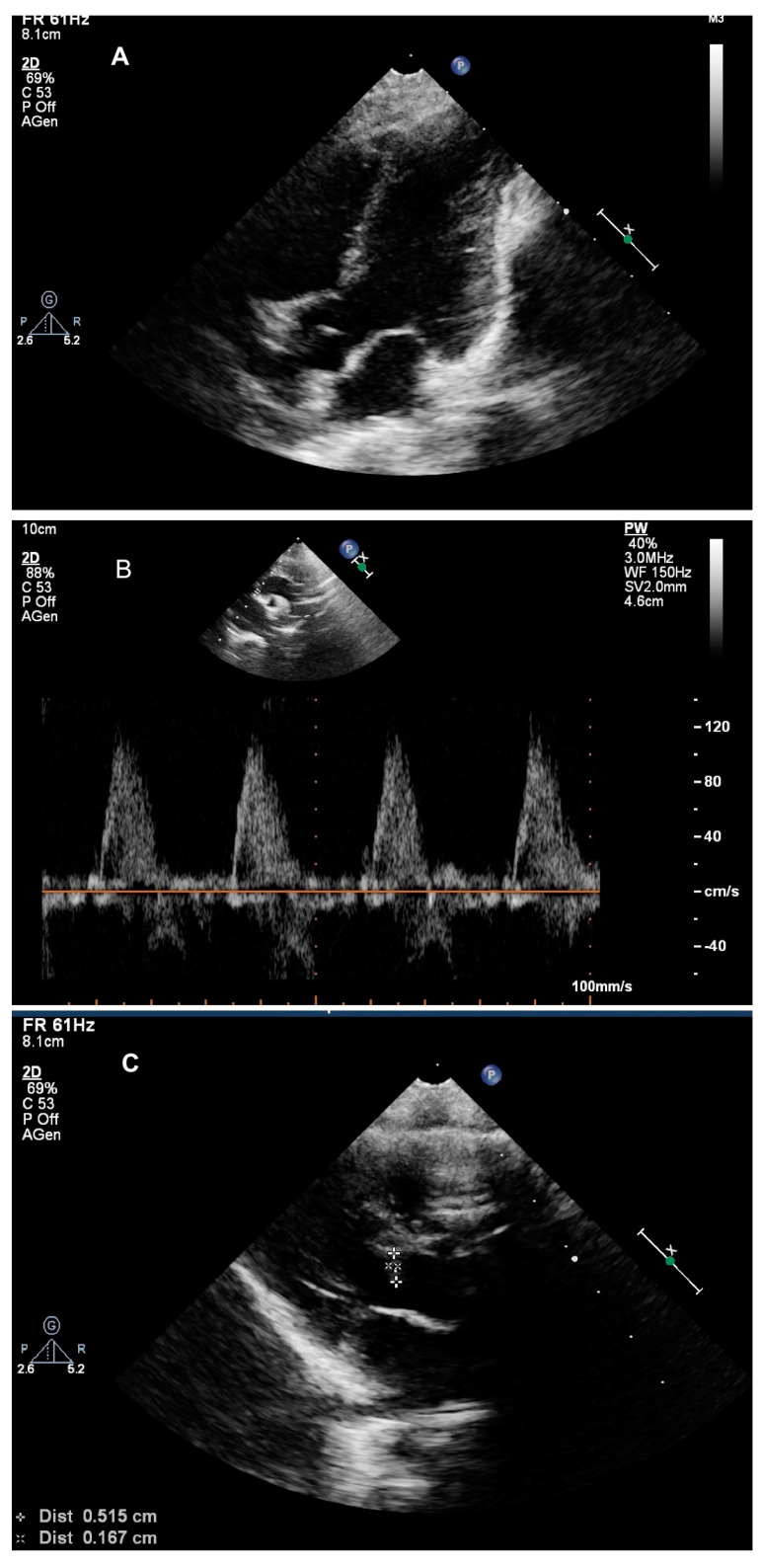
Echocardiogram after 1 week of Everolimus treatment. Panel (**A**)**:** Disappearance of the left ventricle rhabdomyomas; Panel (**B**)**:** Normal transaortic gradient; Panel (**C**): Marked reduction of the subaortic mass.

After 1 week, the serum Everolimus level was 4.4 ng/mL. The infant did not suffer any collateral effects, weight gain was steady, ECG was always normal, without arrhythmia. The cardiac masses continued to shrink with only a slightly elevated transaortic gradient (25/11 mmHg), Figure 2. After only 2 weeks, the transaortic velocity was normal. The masses continued to shrink over the next few months, even though the plasmatic dosage of Everolimus was always at the inferior level of around 3 ng/mL. The child suffered no collateral effects (no major infections, no aftae, normal blood cell count) and neurological evaluations were always normal. 

After 6 months of therapy, an echocardiogram revealed that the mass in the interventricular septum and the masses attached to the mitral chordae were not visible, the subaortic mass was 5 × 2 mm, blood passage was normal, as were the transaortic gradients. The right intraventricular mass had almost completely disappeared. Thus, the Everolimus therapy was discontinued.

Three weeks later, at the ultrasound evaluation the subaortic mass was found to be stable, with normal gradients. The right intraventricular mass and the ones attached to the mitral chordae were visible, but small and not hemodynamically significant. Therefore, we decided not to begin therapy again and planned a monthly echocardiographic follow-up. The baby was asymptomatic, in good clinical conditions, with normal weight gain, and had never contracted infectious diseases. 

## 3. Discussion

CR is the most common primary cardiac pediatric tumor and is a major criterion for TSC diagnosis [1,3]. Most patients with CRs and TSC are asymptomatic, and tumors slowly and spontaneously regress in the first 2 years of life in half of the cases. Some patients become symptomatic and require treatment, with symptoms ranging from arrhythmia and intracardiac blood flow obstruction to congestive heart failure, depending on the size, number, and location of the tumor/s [1,3]. 

The current standard therapy for symptomatic patients is supportive care (diuretics, intravenous inotropic drugs, antiarrhythmics) [1,4]. Surgery is recommended in severe cases, for large tumors that obstruct the outflow tracts of the great arteries or for arrhythmias with a very difficult medical management (generated by infiltration of the tumor and which compromise the nervous conduction) [1,4]. Cardiac surgery involves a number of potential complications such as difficulty in resecting all the tumors, long stays in the intensive care unit, and a high risk of mortality. Moreover, in some cases, considering the size, location, and relation with coronary arteries or intracardiac valves, tumors may be inoperable. Patients with multiple and inoperable CRs may be candidates for the Everolimus treatment. 

Everolimus, a mammalian target of the rapamycin inhibitor drug, has the ability to reduce hamartomas, correcting the specific molecular defect that causes TSC. Everolimus has demonstrated a remarkable suppression effect in children with TSC at doses of 4.7–5.6 mg/m^2^/day and serum trough levels of 5–15 ng/mL [22,23]. 

We described the use of Everolimus in a neonate with multiple biventricular CRs, in which a low-dose drug treatment was successfully adopted, maintaining a minimum therapeutic range.

In the literature, Everolimus therapy in CRs associated with TS has been described in 25 cases occurring in the neonatal period (Table 1). The dosage reported ranged between 0.1–0.5 mg/day although an optimal dosage for neonates is still unknown. As described in Table 1, an initial low dose (0.08–0.1 mg/day) of Everolimus was adopted in only nine neonates. A significant reduction was evident as early as 4 weeks after starting the treatment. Similarly to reports from Chang et al. [15], in our case, the cardiac masses rapidly diminished and after only 5 days of therapy they became clinically insignificant. Of these nine neonates, in seven cases a therapeutic range of 5–15 ng/mL was maintained. In two cases, as in our neonate, a minimum therapeutic range of 3–8 ng/mL was adopted, reducing the risk of toxicity.

Everolimus toxicity occurs in children with TSC less frequently and less severely than in oncologic patients, probably since this drug is a monotherapy for TCS, whereas it is part of a combined treatment for cancer [5].

Adverse events were described in 5/25 (20%) neonates treated with Everolimus, including viral pneumonia (adenovirus and chickenpox) and transient coagulopathy, requiring therapy to be suspended for 1 week in one case. In only one complicated case, a low-dose treatment was used (Table 1). Conversely, our baby was successfully treated, with an absence of any collateral effects, and his weight gain and somatic growth remained within the normal range. On the basis of the literature data and our own experience, a dose of 0.1 mg/kg/day could be a reasonable start for the treatment with Everolimus in neonates.

## 4. Conclusions

In this paper, we show that a low Everolimus dose may be useful to treat CRs in TSC, without any adverse effects and with excellent response to therapy. The extension of Everolimus use for a low-dose treatment of cardiac lesions with a high risk of decompensation, could be considered during the neonatal period.

## Figures and Tables

**Table 1:** Characterization of patients with cardiac rhabdomyomas treated with low doses of Everolimus in the neonatal period.

First Author, Year	Age at Treatment	Clinical Presentation	Echographic Data at Diagnosis	Everolimus Dose	Target Drug Levels	Reported Evolution	Adverse Effects	Follow Up
LOW-DOSE TREATMENT (≤0.1 mg/die)								
Garg A et al., 2019	4th day of life	Prenatal diagnosis of CRs At birth: Pre-excitation with ventricular tachycardia	Multiple CRs (major in RV free wall)	0.08 mg/die	3–7 ng/mL	Improvement of ventricular tachycardia after 4 days of treatment	None	Not reported
Castro-Monsalve J et al., 2018	Not reported	Cardiac arrest	Multiple CRs on interventricular septum with LVOTO	0.1 mg/die	5–8 ng/mL	60% reduction of CR at 31days—resolution at 4 weeks	None	Not reported
Aw F et al., 2016	Average: 6.5 days (2–20 days)	LVOTO 3rd case: Subependymal lesion	1st case: LVOTO e RVOTO 2nd case: Multiple CRs—major subaortic 3rd case: Multiple CRs 4th case: CRs with LVOTO	0.1 mg/die	5–15 ng/mL	50% reduction of CRs in 36 days	Not reported	Treatment: Average 73 days (34–138 days)
Goyer I et al., 2015	Three cases: First week of life	LVOTO In one case: Subependymal astrocytoma	1st case: Multiple CRs (n 6) 2nd case: Multiple CRs with LVOTO 3rd case: Multiple CRs	0.1 mg/die	5–15 ng/mL	Regression at 1 month	Viral infection in one case	Check Everolimus plasmatic concentration every 4 days at the beginning
DOSE TREATMENT (>0.1 mg/die)								
Dhulipudi B et al., 2019	Average: 5 days (1–90 days)	Five case reports describing cardiovascular failure due to outlet obstruction	1st case: CR 24 mm at right outlet 2nd case: CR 13 mm in right atrium with superior vena cava obstruction 3rd case: CR 12 mm with LVOTO 4th and 5th case: Multiple CRs in right ventricle	4.5 mg/m^2^/week divided in daily doses	5–15 ng/mL	CR regression in 6.1 +/− 5.1 months	Chickenpox infection	Not reported
Shibata Y et al., 2019	4th day of life	Prenatal diagnosis of CR	Multiple CRs with LVOTO	Initial dose: 0.4 mg/die, reduced to 0.1 mg/die at day 10	5–15 ng/mL	Reduction of CR after 7 days of therapy	Coagulopathy at day 3 of therapy	Not reported
Martinez-Garcia A et al., 2018	36 days of life	Prenatal diagnosis of multiple CRs	Multiple CRs—major in left ventricle with LVOTO	0.25 mg twice per day—twice per week	Not indicated	Improvement after 2 weeks of treatment	None	Not reported
Chang JS et al., 2017	Not reported	Prenatal diagnosis of CRs In one case: Seizures	1st case: CRs in both ventricles 2nd case: CR in LV with LVOTO 3rd case: Multiple CRs in left ventricle	0.3–0.67 mg/m^2^/die	3–7 ng/mL	Regression in 2 months	1st case: Viral pneumonia 3rd case: Growth failure	Not reported
Colaneri M et al., 2016	Not reported	Prenatal diagnosis of CRs At birth: Hypoplastic left heart syndrome	Multiple CRs—major on left ventricle free wall	0.25 mg/die	5–15 ng/mL	After 10 weeks: 80% reduction of CRs	None	Stop treatment 11 weeks post treatment
Bornaun H et al., 2016	Not reported	Cardiovascular failure due to LVOTO	CR with LVOTO	0.5 mg/die twice per week	2.6–6.1 ng/mL	Regression of lesion in 4 weeks	TG and cholesterol levels increased. Change in lymphocyte subgroups	Stopped therapy after 7.5 months of treatment
Choudhry S et al., 2015	2 weeks of life	Cardiac and cerebral lesions	Multiple RCs	Not reported	Not reported	CR regression in 1 month	Not reported	Not noted
Wagner R et al., 2015	Not reported	At birth: Heart murmur	Multiple CRs—major on LVOTO	Starting dose 1.5 mg/m^2^/die, reduced to 1 mg/m^2^/die at 4th day	5–15 ng/mL	Progressive reduction of RCs	Slightly elevated triglycerides and transitory lymphopenia	Stop therapy at 19 days post treatment
Dogan V et al., 2015	2 days	Heart murmur and cardiovascular failure	CRs with LVOTO	0.25 mg twice per day—twice per week	5–15 ng/mL	Progressive reduction of RCs	Not reported	Stop therapy at 3 months
Oztunc F et al., 2014	First week of life	Supraventricular tachycardia	Multiple CRs (interventricular sept—anterior wall RV)	0.25 mg twice per day—twice per week	Not noted	Supraventricular tachycardia resolution on day 8. Regression of RCs at 15 days	None	Not reported

CR: Cardiac rhabdomyoma; LVOTO: Left ventricular outflow tract obstruction; RVOTO: Right ventricular outflow tract obstruction; RV: Right ventricle.

## Data Availability

All data are reported in the case.
